# Association between psoriasis and vitamin D: Duration of disease correlates with decreased vitamin D serum levels: An observational case-control study

**DOI:** 10.1097/MD.0000000000011185

**Published:** 2018-06-22

**Authors:** Angela Filoni, Michelangelo Vestita, Maurizio Congedo, Giuseppe Giudice, Silvio Tafuri, Domenico Bonamonte

**Affiliations:** a Section of Dermatology, Department of Biomedical Science and Human Oncology; b Unit of Plastic and Reconstructive Surgery, Department of Emergency and Organ Transplantation; c Section of Dermatology, Vito Fazzi Hospital, Piazza Filippo Muratore, Lecce; d Section of Hygiene, Department of Biomedical Science and Human Oncology, University of Bari, Piazza Giulio Cesare, Bari, Italy.

**Keywords:** association, psoriasis, psoriasis duration, serum levels, vitamin D

## Abstract

Recent literature has focused on the association of psoriasis with lower than normal or highly deficient vitamin D blood levels.

To investigate the controversial association between psoriasis and vitamin D levels.

From 2012 to 2014, 561 subjects were assessed, of which 170 had psoriasis, 51 had an autoimmune bullous, and 340 were healthy patients. Anagraphical data, 25(OH)D blood levels, and seasons of vitamin D levels assessments were recorded for each group.

Vitamin D levels were significantly different among the 3 groups (*K* = 151.284; *P = *.0001). Psoriatic patients had significantly lower serum levels of 25(OH)D (21.8 ng/mL) than healthy controls (34.3 ng/mL) (chi-square = 11.5; *P = *.0007). Patients with bullous diseases showed the lowest vitamin D mean values (18.2 ng/mL). The linear multiple regression model showed 25(OH)D levels to be influenced by age, season of blood vitamin D levels assessment, and psoriasis duration.

These results confirm the reduced vitamin D levels in psoriatic patients when compared to healthy controls, and provide new evidence regarding the association of vitamin D levels and psoriasis duration. The limits of our study include its observational nature and the small number of patients undergoing biological immunosuppressive therapies.

**Key points**

- The association between psoriasis and vitamin D levels is controversial, although well-documented.
- Our novel findings indicate that vitamin D levels correlate with psoriasis duration.

## 1 Introduction

Psoriasis is a chronic auto-inflammatory disease involving the innate and acquired immunologic systems as well as keratinocyte differentiation.^[1]^

Vitamin D, as a hormone, performs different tasks besides being involved in calcium-phosphorus metabolism. In fact, vitamin D receptors and CYP271B (enzyme responsible for 25-hydroxyvitamin D (25(OH)D) synthesis) are present in several different tissues.^[2]^ Plasma 25(OH)D concentration is considered by some authors as the best clinical indicator of vitamin D status, reflecting the combined contributions of cutaneous synthesis and dietary intake of vitamin D.^[3]^

Recent literature has focused on the association of several dermatological conditions, such as atopic dermatitis^[4]^ and psoriasis,^[5–7]^ with lower than normal or highly deficient vitamin D blood levels. The definition of vitamin D deficiency has been much debated. Most agree that a serum level of 25(OH)D below 20 ng/mL is an indication of vitamin D deficiency; whereas vitamin D insufficiency is defined as a serum level of 25(OH)D ranging from 20 to 30 ng/mL.^[8]^ These values do not necessarily present with overt clinical symptoms, and have recently become a concern due to their prevalence; mainly, associated with other clinical conditions.^[8]^ In this work, we further investigate the association between psoriasis and vitamin D levels, trying to clarify the currently controversial available data.^[9]^

## 2 Materials and methods

From 2012 to 2014, 561 consecutive subjects were assessed and divided into 3 groups: psoriasis patients, bullous dermatoses patients (as a positive control group), and healthy controls (as a negative control group). Psoriasis and bullous disease patients were recruited from the Units of Dermatology of the University of Bari and Lecce “Vito Fazzi” Hospital, while controls were recruited from among the elective nononcological patients from the Unit of Plastic Surgery of Bari. Anagraphical data, 25(OH)D blood levels, season of serum vitamin D levels assessment, type of job (indoor or outdoor), and menopause status (yes or no) were recorded for each group. These data were collected since they may have contributed to the measured vitamin D levels. In the psoriatic patients, psoriasis duration, severity (mild Psoriasis Area and Severity Index (PASI) <7, moderate PASI 7–12 or severe PASI >12), family history, clinical presentation, and previous and current therapies for psoriasis were also recorded. Patients of all groups were recruited if aged >18 years. Patients with concomitant chronic inflammatory disease or malignancy, and those receiving therapeutic interventions that might influence vitamin D status, including bisphosphonates, systemic corticosteroids, vitamin D, and calcium supplements were excluded. Psoriasis patients undergoing phototherapy or topical vitamin D therapies were also excluded. All included subjects were Caucasian, white-skinned, and from the same geographical area to avoid differences in sun exposure and vitamin D diet intake. All patients were required to sign an informed consent. Data were collected and analyzed anonymously, after insertion in a password-protected database.

### 2.1 Statistical analysis

Data were inserted into a FileMaker Pro database and analyzed with Microsoft Office Excel e STATA MP12 software. Continuous variables were expressed as average ± standard deviation and range, while categorical variables were expressed as proportions. The Ladder of powers test was used to assess the distribution of variables. To assess the determinants of vitamin D values, a multiple linear regression model was designed, using vitamin D levels as outcomes and sex, age, job (indoor/outdoor), menopause status, disease severity and duration, psoriasis clinical type including psoriatic arthritis, current and previous treatment for psoriasis, and season of vitamin D blood testing as determinants. To compare the distribution of the categorical variables in the 3 different groups, the chi-square test was used. To compare quantitative continuous variables, the Kruskal–Wallis test for independent samples with non-normal distribution was used.

A value of *P < .*05 was considered statistically significant for each test.

## 3 Results

The study population included 561 subjects, of which 170 had psoriasis (40 had psoriatic arthritis), 51 had an autoimmune bullous disease (36 had pemphigus and 15 had pemphigoid), and 340 were healthy patients. Table 1 summarizes the characteristics of the 3 studied patient groups, while Table 2 reports the intrinsic characteristics of the psoriasis group.

**Table 1:**
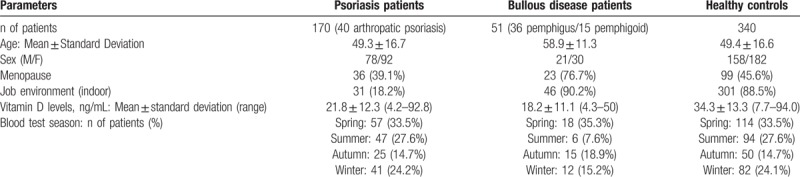
Compared anagraphical and demographic characteristics of the psoriasis, bullous disease and control patients.

**Table 2:**
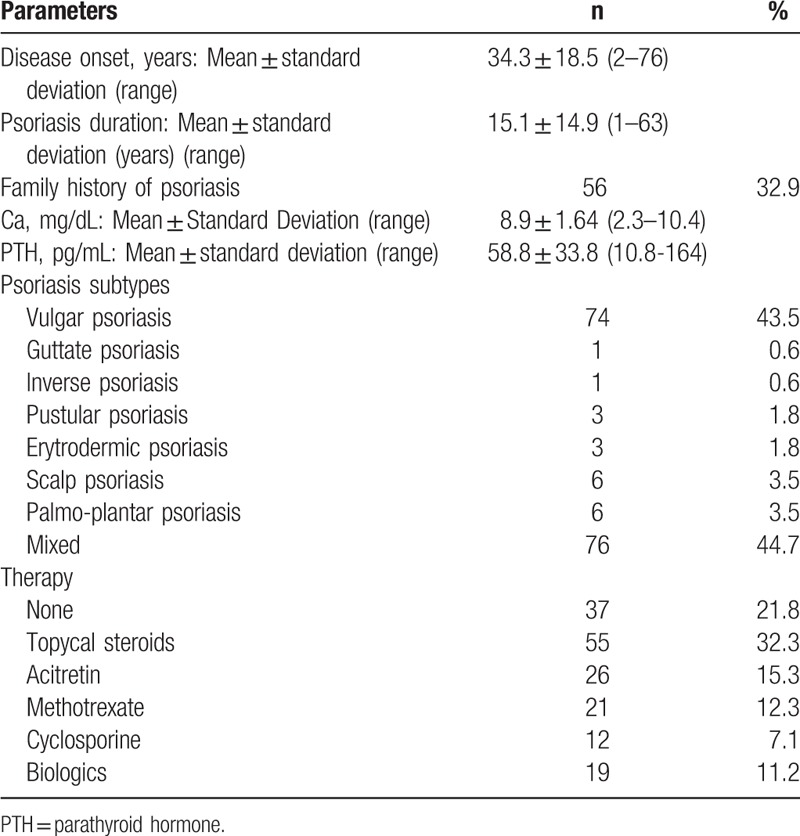
Disease-related characterisitcs of the psoriasis patients group, including psoriasis duration, subtype and administered therapy.

Patient age was significantly different among the 3 groups (*K* = 13.609; *P = *.0011), but no significant difference occurred between the psoriatic and healthy controls (*K* = 4.5; *P > *.05).

**Figure 1:**
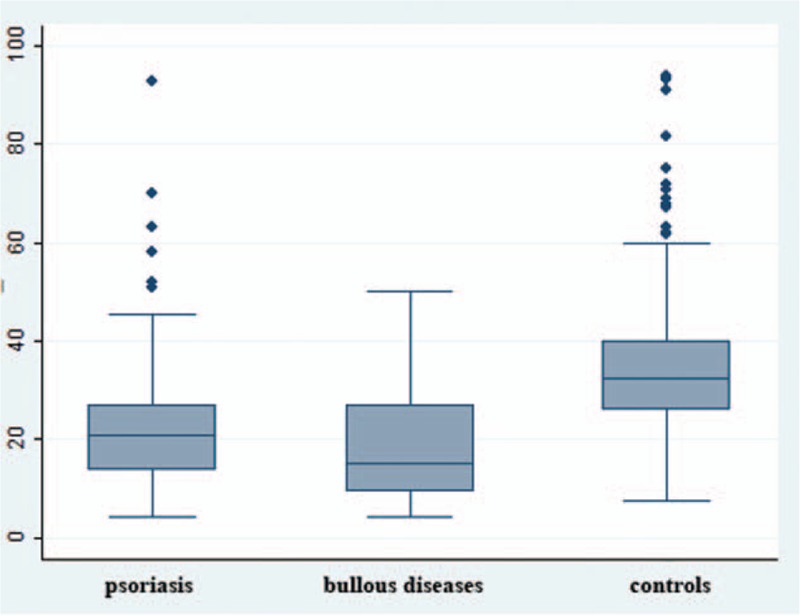
Mean vitamin D values in the 3 groups (*K* = 151,284; *P = *,0001).

Vitamin D levels were significantly different among the 3 groups (*K* = 151.284;*P = *.0001, Fig. 1). Psoriatic patients had significantly lower serum levels of 25(OH)D (21.8 ng/mL) than the healthy controls (34.3 ng/mL) (*K* = 11.5; *P = *.0007). 45.8% (n 78) and 38.9% (n 66) of psoriasis patients were vitamin D deficient (<20 ng/mL) and insufficient (20–30 ng/mL). Patients with bullous diseases showed the lowest vitamin D mean values (18.2 ng/mL).

The linear multiple regression model revealed 25(OH)D levels to be influenced by age, season of blood vitamin D levels assessment, and psoriasis duration. No other variables showed any influence on 25(OH)D levels (Table 3).

**Table 3:**
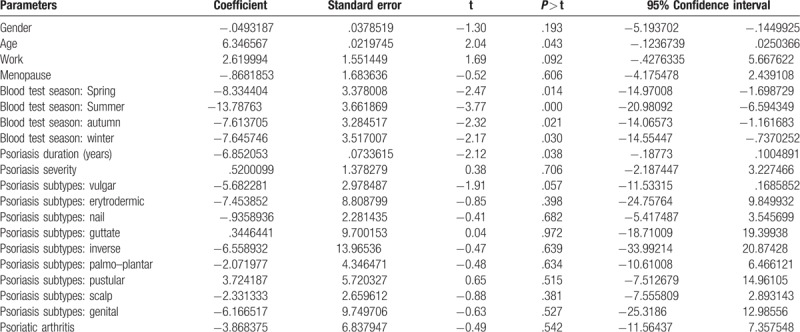
Linear regression model: analysis of determinants of vitamin D serum levels.

## 4 Discussion

Data regarding the association between psoriasis and vitamin D are presently controversial. Initial observations demonstrated significantly lower vitamin D levels in psoriatic patients when compared to healthy controls.^[10,11]^ Later studies did not confirm these results.^[12–14]^ However, many factors can affect vitamin D deficiency and insufficiency prevalence, such as race, ultraviolet radiation exposure, and vitamin D dietary intake;^[15]^ therefore, caution is needed when comparing the different studies and results.

In our observation, the logistic regression analysis showed how vitamin D serum levels related with patient age, season of blood vitamin D levels assessment, and psoriasis duration. The latter finding has only been evidenced once by a recent study,^[6]^ and therefore, constitutes a relatively novel finding in international literature.

It is possible that patients with psoriasis may have lower vitamin D levels than the ordinary population due to a series of factors. In fact, low 25(OH)D levels can either represent the cause or consequence of psoriasis, resulting from lack of sun exposure, from frequent use of drugs that interfere with 25(OH)D metabolism (such as gluco-corticoids and immunosuppressive agents), or from low 25(OH)D intake.^[16,17]^

It is a known fact that patients with psoriasis, except those undergoing phototherapy, tend to keep their affected areas covered. This attitude, prolonged during the years, could lead to decreased UV exposure with consequent reduced vitamin D levels. Therefore, those affected by long-time psoriasis, and not undergoing phototherapy, could possibly be more prone to vitamin D reduced serum levels.^[17]^

Another explanation lies in a possible pathogenic role of vitamin D in psoriasis. Psoriasis is considered to be a Th1-Th17-Th22-based autoimmune inflammatory disease that involves the innate and acquired immunity.^[16]^ A low 25(OH)D status is known to be associated with an increased risk of developing Th1-mediated autoimmune diseases. Moreover, a suppressive or inhibiting effect of vitamin D has been demonstrated in many autoimmune diseases through receptors on activated T lymphocytes, including rheumatoid arthritis, type 1 diabetes, inflammatory bowel disease, and multiple sclerosis.^[18,19]^

Furthermore, vitamin D has been shown to regulate keratinocyte differentiation.^[20]^ In fact, at physiological concentrations, vitamin D promotes keratinocyte growth and development, protecting them from early apoptosis. Vitamin D also modulates the expression of K1 and K10 in the stratum spinosum; 2 important keratins whose expression in psoriatic skin is altered.^[21,22]^ Other studies have further supported this concept with immunohistochemical and biochemical assays, demonstrating the antiproliferative and pro-differentiating effects in epidermal keratinocytes when exposed to 1,25(OH)2D3.^[23]^ Finally, vitamin D has been shown to normalize the distribution of integrins, which are commonly altered in psoriatic skin, such as CD26 and ICAM-1, in the dermal–epidermal junction.^[18,24]^

Despite these evidences, the precise role of vitamin D in psoriasis is still unclear.^[25,26]^ but one could suppose low vitamin D levels could favor psoriasis chronicization, thorough a reduced inhibitory effect on T lymphocytes. However, of note, the correlation between psoriasis severity and vitamin D levels is also controversial, and no correlation has been discovered with psoriasis duration prior to this observation. Finally, psoriatic patients are known to suffer from metabolic syndrome, leading to a series of comorbidities, including an increased Body Mass Index and obesity.^[27]^ The latter translates in increased fat deposits, in which vitamin D tends to accumulate; consequently, reducing circulating bioavailable levels.^[28,29]^ In fact, decreased circulating vitamin D observed in subjects with severe psoriasis has been tentatively explained via the liposolubility of vitamin D and its reduced bioavailability in patients with a high percentage of fat content.^[30,31]^ Similarly, in another study, psoriasis patients with low serum vitamin D showed more comorbidities, especially in terms of a higher prevalence of increased BMI or dyslipidemia.^[32]^

To conclude, our results confirmed reduced vitamin D levels in psoriatic patients when compared to healthy controls, which provides new evidence regarding the association of vitamin D levels and psoriasis duration. The limitations of our study include its observational nature and the small number of patients undergoing biological immunosuppressive therapies. Further observational and randomized-controlled clinical trials are warranted to confirm our results.

## Author contributions

**Conceptualization:** Angela Filoni, Michelangelo Vestita, Maurizio Congedo, Domenico Bonamonte.

**Data curation:** Angela Filoni, Michelangelo Vestita, Maurizio Congedo, Silvio Tafuri, Domenico Bonamonte.

**Formal analysis:** Angela Filoni, Maurizio Congedo, Silvio Tafuri.

**Funding acquisition:** Angela Filoni.

**Investigation:** Angela Filoni, Michelangelo Vestita, Maurizio Congedo, Domenico Bonamonte.

**Methodology:** Angela Filoni, Michelangelo Vestita, Silvio Tafuri.

**Project administration:** Angela Filoni, Michelangelo Vestita, Domenico Bonamonte.

**Resources:** Silvio Tafuri.

**Software:** Silvio Tafuri.

**Supervision:** Michelangelo Vestita, Giuseppe Giudice, Domenico Bonamonte.

**Validation:** Michelangelo Vestita, Giuseppe Giudice, Domenico Bonamonte.

**Visualization:** Michelangelo Vestita, Domenico Bonamonte.

**Writing – original draft:** Angela Filoni, Michelangelo Vestita.

**Writing – review & editing:** Angela Filoni, Michelangelo Vestita, Giuseppe Giudice.
